# A New HPLC-UV Method Using Hydrolyzation with Sodium Hydroxide for Quantitation of *Trans*-*p*-Hydroxycinnamic Acid and Total *Trans*-*p*-Hydroxycinnamic Acid Esters in the Leaves of *Ligustrum robustum*

**DOI:** 10.3390/molecules28145309

**Published:** 2023-07-10

**Authors:** Shi-Hui Lu, Xiao-Na Liang, Xiao-Jin Nong, Ran Chen, Xiu-Xia Li

**Affiliations:** 1 College of Pharmacy, Youjiang Medical University for Nationalities, Baise 533000, China; 20221001046@stu.ymun.edu.cn (X.-N.L.); n15777644073@163.com (X.-J.N.); 2 Guangxi Database Construction and Application Engineering Research Center for in Tracorporal Pharmacochemistry of TCM, Baise 533000, China; 3 Key Laboratory of Youjiang Basin Characteristic Ethnic Medicine Research in Guangxi, Baise 533000, China; 4 Institute of Life Science, Youjiang Medical University for Nationalities, Baise 533000, China; ran625@ymun.edu.cn; 5 Nursing School, Youjiang Medical University for Nationalities, Baise 533000, China

**Keywords:** *trans*-*p*-hydroxycinnamic acid, esters, *Ligustrum robustum*, HPLC-UV, hydrolyzation, sodium hydroxide, quantification

## Abstract

*Trans*-*p*-hydroxycinnamic acid and its esters in the leaves of *Ligustrum robustum* might be a new resource to prevent diabetes and its complications. In the present study, a new HPLC-UV method using hydrolyzation with sodium hydroxide for quantitation of *trans*-*p*-hydroxycinnamic acid and total *trans*-*p*-hydroxycinnamic acid esters in the leaves of *L. robustum* was developed, since it was difficult and troublesome to analyze no less than 34 *trans*-*p*-hydroxycinnamic acid esters by usual HPLC. The extract of *L. robustum* was hydrolyzed with sodium hydroxide at 80 °C for 2 h, and then, hydrochloride was added. HPLC analysis was performed in reverse phase mode using a C-18 column, eluting with methanol-0.1% acetic acid aqueous solution (40:60, *v*/*v*) in isocratic mode at a flow rate of 1.0 mL·min^−1^ and detecting at 310 nm. The linear range for *trans*-*p*-hydroxycinnamic acid was 11.0–352.0 μg·mL^−1^ (*r*^2^ = 1.000). The limit of detection and limit of quantification were 2.00 and 6.07 μg·mL^−1^, respectively. The relative standard deviations of intra-day and inter-day variabilities for *trans*-*p*-hydroxycinnamic acid were less than 2%. The percentage recovery of *trans*-*p*-hydroxycinnamic acid was 103.3% ± 1.1%. It is the first HPLC method using hydrolyzation for quantification of many carboxylic esters. Finally, the method was used successfully to determine *trans*-*p*-hydroxycinnamic acid and total *trans*-*p*-hydroxycinnamic acid esters in various extracts of the leaves of *L. robustum*. The 60–70% ethanol extracts of *L. robustum* showed the highest contents of free *trans*-*p*-hydroxycinnamic acid (3.96–3.99 mg·g^−1^), and the 50–80% ethanol extracts of *L. robustum* displayed the highest contents of total *trans*-*p*-hydroxycinnamic acid esters (202.6–210.6 mg·g^−1^). The method can be applied also to the quality control of the products of *L. robustum*.

## 1. Introduction

*Ligustrum robustum* (Roxb.) Blume, a plant of Oleaceae, is distributed widely in Southwest China, Burma, Vietnam, India, and Cambodia [1]. The leaves of *L. robustum* have been used as Ku-Ding-Cha, a functional tea to clear heat and remove toxins, in Southwest China for near 2000 years [2,3]. Additionally, *L. robustum* was served as a folk medicine to deal with diabetes, obesity, hypertension, and so on [3,4].

**Figure 1:**
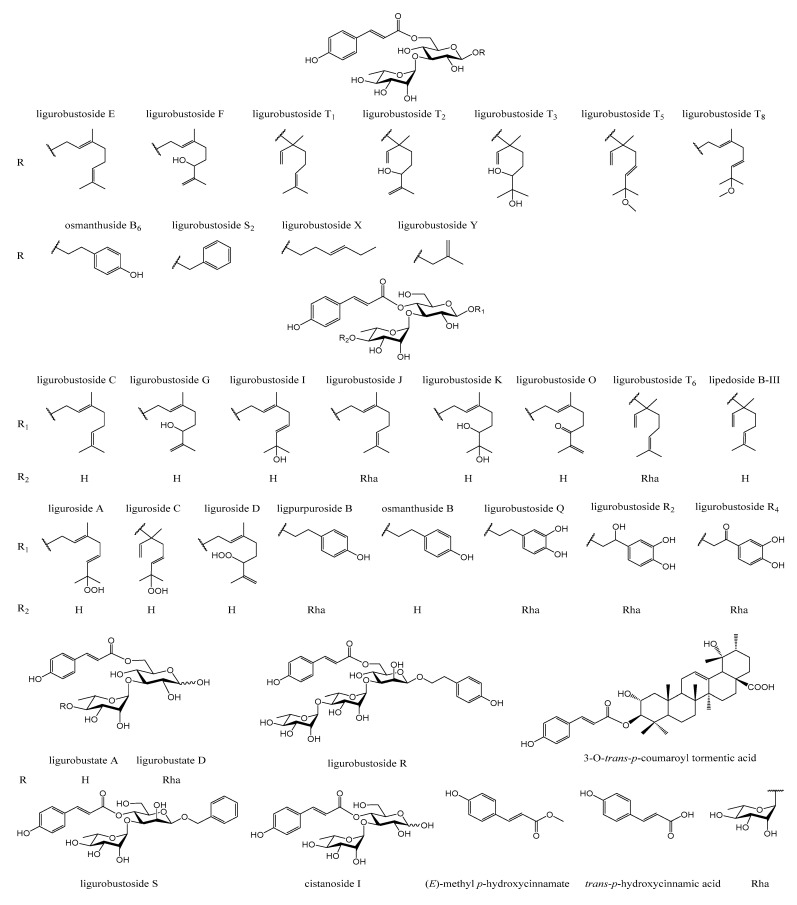
Structures of *trans*-*p*-hydroxycinnamic acid and its esters isolated from the leaves of *L. robustum*.

In the previous phytochemical studies [2,5,6,7,8,9,10,11,12,13,14,15,16,17], about 90 chemical constituents, including *trans*-*p*-hydroxycinnamic acid, 34 *trans*-*p*-hydroxycinnamic acid esters (Figure 1), flavonoid glycosides, lignan glycosides, and other compositions, were isolated and identified from the leaves of *L. robustum*.

In our previous biological investigations [14,15,16,17], the chemical compositions isolated from the leaves of *L. robustum* were tested, and the results showed that (1) *trans*-*p*-hydroxycinnamic acid displayed stronger *α*-glucosidase inhibitory activity than the positive control acarbose; (2) *trans*-*p*-hydroxycinnamic acid and several *trans*-*p*-hydroxycinnamic acid esters displayed no weaker fatty acid synthase (FAS) inhibitory activities than the positive control orlistat; (3) *trans*-*p*-hydroxycinnamic acid and a lot of *trans*-*p*-hydroxycinnamic acid esters revealed stronger 2,2′-azino-bis(3-ethylbenzthiazoline-6-sulphonic acid) ammonium salt (ABTS) radical scavenging effects than the positive control L-(+)-ascorbic acid and revealed moderate α-amylase inhibitory activities. What is more, *trans*-*p*-hydroxycinnamic acid esters might be hydrolyzed with catalysis of carboxylesterase or gastric acid in vivo [18,19] and release *trans*-*p*-hydroxycinnamic acid, meaning that *trans*-*p*-hydroxycinnamic acid esters are the prodrugs of *trans*-*p*-hydroxycinnamic acid. In addition, it was reported that natural products with inhibitory activities on α-glucosidase, α-amylase, and FAS as well as an antioxidant effect might be a new resource to prevent diabetes and its complications, which affected nearly 10.5% of the population in the world and led to serious mortality [17]. Therefore, *trans*-*p*-hydroxycinnamic acid and its esters isolated from *L. robustum* might be a novel resource for preventing diabetes and its complications.

It was significant to analyze the contents of *trans*-*p*-hydroxycinnamic acid and its esters in the leaves of *L. robustum*. Some HPLC methods for the simultaneous determination of *trans*-*p*-hydroxycinnamic acid and several other compositions have been published [20,21,22], but the quantitative analytical method for *trans*-*p*-hydroxycinnamic acid and its esters in the leaves of *L. robustum* has not been reported so far. HPLC is a common and accurate method to simultaneously analyze several compositions [20,21,22], but it was difficult and troublesome to determine 34 *trans*-*p*-hydroxycinnamic acid esters by usual HPLC. In addition, there might be other unknown *trans*-*p*-hydroxycinnamic acid esters in the leaves of *L. robustum*. In the present study, thus, a new HPLC-UV method using hydrolyzation with sodium hydroxide for quantitation of *trans*-*p*-hydroxycinnamic acid and total *trans*-*p*-hydroxycinnamic acid esters in the leaves of *L. robustum* was developed and validated. Moreover, the proposed method was applied successfully to the determination and comparison of *trans*-*p*-hydroxycinnamic acid and total *trans*-*p*-hydroxycinnamic acid esters in various extracts of the leaves of *L. robustum*.

## 2. Results and Discussion

### 2.1. Method Development

#### 2.1.1. Optimization of the Chromatographic Conditions

**Figure 2:**
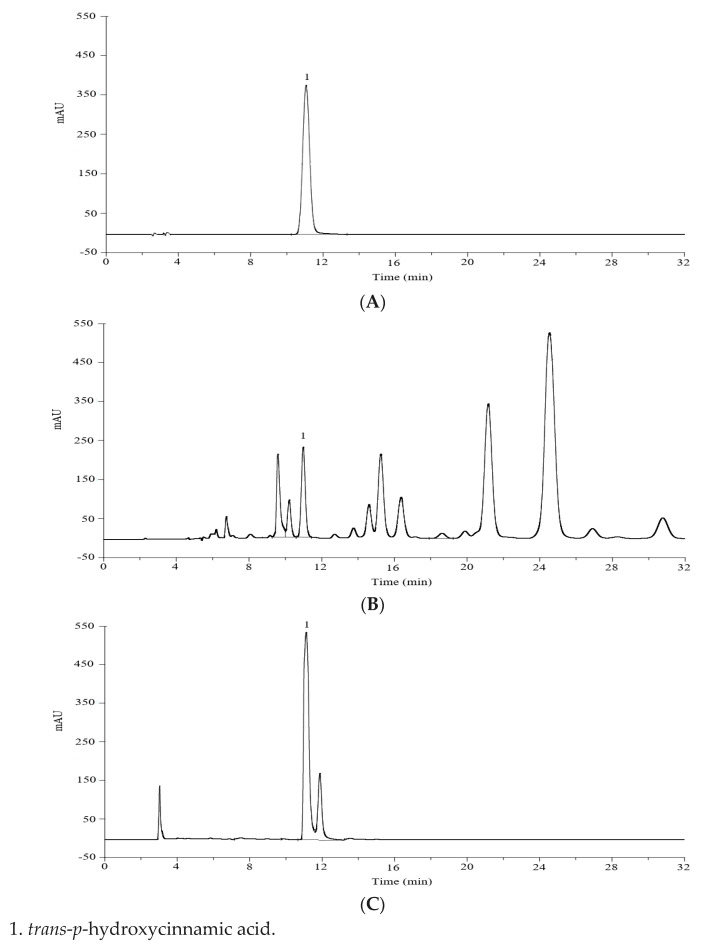
HPLC chromatograms of *trans*-*p*-hydroxycinnamic acid (**A**), the original extract of *L. robustum* (**B**), and the hydrolyzed extract of *L. robustum* (**C**).

The raw powder of the dried leaves of *L. robustum* was extracted with 70% (*v*/*v*) ethanol (25 mL·g^−1^) under reflux for 1 h. And the *trans*-*p*-hydroxycinnamic acid esters in the extracting solution of *L. robustum* were hydrolyzed with sodium hydroxide at 80 °C for 2 h. In order to quantify the free *trans*-*p*-hydroxycinnamic acid in the original extracting solution and the potential *trans*-*p*-hydroxycinnamic acid (including the free *trans*-*p*-hydroxycinnamic acid in the original extracting solution and the *trans*-*p*-hydroxycinnamic acid released from the *trans*-*p*-hydroxycinnamic acid esters) in the hydrolyzed extracting solution of *L. robustum*, several chromatographic items were considered. The first factor was the stationary phase. When using C-18 column (4.6 mm × 250 mm, 5 μm) or C-8 column (4.6 mm × 250 mm, 5 μm), eluting with methanol-0.1% acetic acid aqueous solution (40:60, *v*/*v*) and detecting at 310 nm, the resolution value of *trans*-*p*-hydroxycinnamic acid from other compositions was more than 1.5 and 1.2, respectively. The second factor was the mobile phase. Methanol, acetonitrile, and ultrapure water in different volumes were tried, and methanol–water (40:60, *v*/*v*), without glacial acetic acid, showed acceptable resolution value but with tailed peak. Consequently, glacial acetic acid was added to improve the resolution and peak shape. Furthermore, the UV detector was successfully applied for the detection of the compositions, and the wavelength of 310 nm gave the maximum sensitivity at 30 °C (Supplementary Figure S1). Generally, the optimal HPLC performance (Figure 2) was observed when using C-18 column (4.6 mm × 250 mm, 5 μm), eluting with methanol-0.1% acetic acid aqueous solution (40:60, *v*/*v*) at a flow rate of 1.0 mL·min^−1^, and detecting at 310 nm.

#### 2.1.2. Optimization of the Hydrolyzed Conditions of *Trans*-*p*-Hydroxycinnamic Acid Esters

**Figure 3:**
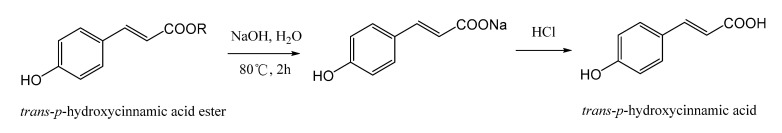
Hydrolyzation of *trans*-*p*-hydroxycinnamic esters.

The hydrolyzed conditions of *trans*-*p*-hydroxycinnamic acid esters in the extracting solution of *L. robustum* were optimized by single factor test with the content of potential *trans*-*p*-hydroxycinnamic acid as the index. The first factor was the catalyst of hydrolyzation. The extracting solution of *L. robustum* was hydrolyzed with different catalysts (hydrochloride, sodium hydroxide) at 80 °C for 2 h; then, the catalyst was neutralized, and *trans*-*p*-hydroxycinnamic acid sodium salt was transformed into *trans*-*p*-hydroxycinnamic acid. The content of potential *trans*-*p*-hydroxycinnamic acid in the hydrolyzed extracting solution was analyzed by HPLC at the optimal chromatographic conditions. As shown in Table 1, the content of potential *trans*-*p*-hydroxycinnamic acid hydrolyzed with sodium hydroxide (66.0 ± 0.5 mg·g^−1^) was higher than those hydrolyzed with hydrochloride (62.0 ± 0.5 mg·g^−1^). The second factor was incubation temperature. The extracting solution of *L. robustum* was hydrolyzed with sodium hydroxide at 30–90 °C for 2 h; then, hydrochloride was added, and *trans*-*p*-hydroxycinnamic acid sodium salt was transformed into *trans*-*p*-hydroxycinnamic acid. The content of potential *trans*-*p*-hydroxycinnamic acid hydrolyzed at 80 °C (66.2 ± 0.5 mg·g^−1^) was the highest (Table 1). The third factor was period of incubation. The extracting solution of *L. robustum* was hydrolyzed with sodium hydroxide at 80 °C for 1–6 h; then, hydrochloride was added, and *trans*-*p*-hydroxycinnamic acid sodium salt was transformed into *trans*-*p*-hydroxycinnamic acid. The content of potential *trans*-*p*-hydroxycinnamic acid hydrolyzed for 2 h (66.0 ± 0.5 mg·g^−1^) was the highest (Table 1). Therefore, the optimal hydrolyzed conditions were as follows: 1 mL sodium hydroxide aqueous solution (1 M) was added into 1 mL the extracting solution of *L. robustum* and incubated at 80 °C for 2 h, and then, 1 mL hydrochloride (1 M) solution was added (Figure 3).

#### 2.1.3. Calculation of the Total Concentration of *Trans*-*p*-hydroxycinnamic Esters in the Extracting Solution of *L. robustum*

The total concentration of *trans*-*p*-hydroxycinnamic esters (C_t_, µmol·mL^−1^) in the extracting solution of *L. robustum* was calculated with the following equation:

C_t_ = C_p_ − C_f_ (1)

C_p_ (µmol·mL^−1^): The concentration of potential *trans*-*p*-hydroxycinnamic acid (molecular weight: 164.16) in the hydrolyzed extracting solution of *L. robustum*. It was calculated as C_p_ (µg·mL^−1^)/164.16.

C_f_ (µmol·mL^−1^): The concentration of free *trans*-*p*-hydroxycinnamic acid in the original extracting solution of *L. robustum*. It was calculated as C_f_ (µg·mL^−1^)/164.16.

Osmanthuside B (molecular weight: 592.59) was considered as a representation of *trans*-*p*-hydroxycinnamic acid esters because of its moderate molecular weight and high content (49.6 mg·g^−1^) in the leaves of *L. robustum*. Consequently, C_t_ (µg·mL^−1^) might be calculated approximatively as C_t_ (µmol·mL^−1^) × 592.59.

In order to overcome some drawbacks of the usual HPLC method, such as the analysis of the compositions lacking chromophore groups, chemical derivatization with appropriate reagents before HPLC-UV analysis is generally used [23]. Therefore, to deal with the difficulty and trouble of the determination of no less than 34 *trans*-*p*-hydroxycinnamic acid esters by usual HPLC, these esters were hydrolyzed with sodium hydroxide before HPLC-UV analysis in the present study. This method was simple and rapid, which greatly simplified the analytical process. Nevertheless, the shortcoming of the method is that the total content of the *trans*-*p*-hydroxycinnamic acid esters is clear, but the specific content of every ester is not clear.

### 2.2. Method Validation

#### 2.2.1. Specificity

The peak of *trans*-*p*-hydroxycinnamic acid (retention time 10.89 ± 0.02 min) was clearly identified in the chromatogram of the standard solution (Figure 2A), while a main peak was also confirmed at the same retention time in the chromatograms of the original extract of *L. robustum* (Figure 2B) and the hydrolyzed extract of *L. robustum* (Figure 2C). The resolutions between *trans*-*p*-hydroxycinnamic acid and other compositions in the real samples were more than 1.5 (Figure 2). In addition, the purity factors of the peak of *trans*-*p*-hydroxycinnamic acid in Figure 2B,C were 978.45 and 951.87, respectively. These data demonstrated that the other components in the real samples hardly interfered with the peak of *trans*-*p*-hydroxycinnamic acid, reflecting the specificity of the method.

#### 2.2.2. Linearity and Calibration Curve

The calibration curve for *trans*-*p*-hydroxycinnamic acid was obtained by external standard method, using six concentrations of the standard, with three injections per concentration (Supplementary Table S1). The chromatogram peak areas were plotted against the corresponding concentrations of the standard solutions to establish the calibration curve (Supplementary Figure S2), and linear regression equation was calculated by the least squares method. This HPLC method showed linear regression at concentrations from 11.0 to 352.0 μg·mL^−1^ , and the determination coefficient (*r*^2^) was 1.000 (Table 2), indicating excellent linearity.

#### 2.2.3. Limit of Detection and Limit of Quantification

The standard deviation of the Y-intercept in the regression equation was used as the residual standard deviation [24]. Consequently, the limit of detection (LOD) and limit of quantification (LOQ) were 2.00 and 6.07 μg·mL^−1^, respectively (Table 2).

#### 2.2.4. Precision

In order to validate the precision of this HPLC-UV method, the hydrolyzed extract of *L. robustum* was determined at three different concentrations, and the relative standard deviations (RSD) were calculated from the results of repeated measurements for each concentration. The measurement was performed three times in the same day to obtain the intra-day variability and carried out three times in three different days to give the inter-day variability. The RSDs of intra-day and inter-day variabilities for *trans*-*p*-hydroxycinnamic acid were less than 2% (Table 3), indicating that the precision of this method was in accordance with the criterion recommended by the International Council for Harmonisation of Technical Requirements for Pharmaceuticals for Human Use (ICH) guidelines [24].

#### 2.2.5. Accuracy

To evaluate the accuracy of this analytical method, a recovery experiment was performed in triplicate at three concentration levels. The extracting solution of *L. robustum*, which was spiked with 50%, 100%, or 150% of its native amount of potential *trans*-*p*-hydroxycinnamic acid, was hydrolyzed with sodium hydroxide. The potential *trans*-*p*-hydroxycinnamic acid in the hydrolyzed solution was analyzed. And the percentage recovery was calculated. As showed in Table 4, the percentage recovery of *trans*-*p*-hydroxycinnamic acid was 103.3% ± 1.1%, indicating that the interference of the small peak overlapping slightly with the *trans*-*p*-hydroxycinnamic acid peak (Figure 2C) was little, and the accuracy of the method was acceptable.

#### 2.2.6. System Suitability Parameters

The soundness of the method was tested by recording the parameters of the system suitability, and the results for *trans*-*p*-hydroxycinnamic acid in Figure 2B,C are presented in Table 5.

#### 2.2.7. Robustness

The robustness is the ability of a method to remain unaffected by slight and deliberate changes in the experimental conditions. The robustness of the analytical method was assessed by testing the influence of slight changes in column temperature (30 ± 5 °C), flow rate (1.0 ± 0.2 mL·min^−1^), mobile phase composition (40% ± 2% methanol), and wavelength (310 ± 2 nm). The RSD of the peak area of *trans*-*p*-hydroxycinnamic acid was found to be in the range of 0.55–0.98% (Table 6). In all cases, the RSD was less than 2%, indicating the robustness of the optimized method [25].

### 2.3. Quantification of Trans-p-Hydroxycinnamic Acid and Total Trans-p-Hydroxycinnamic Acid Esters in Various Extracts of L. robustum

The dried leaves (10.00 g) of *L. robustum* were extracted with 90 mL ethanol aqueous solution (30%, 40%, 50%, 60%,70%, 80%, *v*/*v*) under reflux for 60 min, and the contents of *trans*-*p*-hydroxycinnamic acid and total *trans*-*p*-hydroxycinnamic acid esters in the extracts were determined by the above developed and validated method. The results are presented in Table 7. The 60–70% ethanol extracts of *L. robustum* showed the highest contents of free *trans*-*p*-hydroxycinnamic acid (3.96–3.99 mg·g^−1^), while the 30% ethanol extract of *L. robustum* showed the lowest content of free *trans*-*p*-hydroxycinnamic acid (2.26 mg·g^−1^). In addition, the 50–80% ethanol extracts of *L. robustum* displayed the highest contents of total *trans*-*p*-hydroxycinnamic acid esters (202.6–210.6 mg·g^−1^), while the 30% ethanol extract of *L. robustum* displayed the lowest content of total *trans*-*p*-hydroxycinnamic acid esters (125.8 mg·g^−1^). Taken together, 60–70% ethanol was the optimal extraction solvent for *trans*-*p*-hydroxycinnamic acid and its esters.

### 2.4. Similarity of HPLC Chromatograms

The similarity of the HPLC chromatogram of the original extract of *L. robustum* (Figure 2B) with the HPLC chromatogram of the hydrolyzed extract of *L. robustum* (Figure 2C) was 0.103 (Table 7), which was low because there was only one common peak (*trans*-*p*-hydroxycinnamic acid) in Figure 2B,C.

## 3. Materials and Methods

### 3.1. Chemicals and Reagents

Methanol (HPLC) was afforded by Saimo Fisher Scientific Co., Ltd. (Shanghai, China). Ethanol (AR) was purchased from Chengdu Kelong Chemical Co., Ltd. (Chengdu, China). Glacial acetic acid, sodium hydroxide, and hydrochloride (AR) were acquired from Shanghai Aladdin Biochemical Technology Co., Ltd. (Shanghai, China). The standard of *trans*-*p*-hydroxycinnamic acid (>98% purity) was isolated and identified in our laboratory from the leaves of *L. robustum*, as previously described [14]. Ultrapure water was obtained from an ultra-pure water purifier system (Chengdu Yuechun Scientific Co., Ltd., Chengdu, China).

### 3.2. Plant Material

The leaves of *L. robustum* (Ku-Ding-Cha), which were collected in August 2021 and dried at 100–120 °C for 1 h, were purchased from Junlian Qing-Shan-Lu-Shui Tea Co., Ltd. (Yibin, China). The material was crushed by hand before extraction (≤0.5 cm).

### 3.3. Hydrolyzation of Extracting Solution and Preparation of Solutions

Standard solution of *trans*-*p*-hydroxycinnamic acid: the stock solution of *trans*-*p*-hydroxycinnamic acid was diluted with 40% (*v*/*v*) methanol to obtain 6 standard solutions at 11.0, 22.0, 44.0, 88.0, 176.0, and 352.0 μg·mL^−1^ and filtered through a 0.45 μm PTFE syringe filter (Millipore, Billerica, MA, USA) before HPLC analysis.

Test solution of original extract: the original extracting solution of *L. robustum* was diluted with 40% (*v*/*v*) methanol and percolated using a 0.45 μm PTFE syringe filter prior to HPLC measurement.

Test solution of hydrolyzed extract: 1 mL sodium hydroxide aqueous solution (1 M) and 1 mL the extracting solution of *L. robustum* were mixed and incubated at 80 °C for 2 h [26], and then, 1 mL hydrochloride (1 M) solution was added. After it cooled down to room temperature, the above mixture solution was transferred to 10 mL volumetric flask, and the volume was filled with 40% (*v*/*v*) methanol. The diluted solution was filtered with a 0.45 μm PTFE syringe filter prior to HPLC determination.

### 3.4. HPLC Determination of Trans-p-Hydroxycinnamic Acid and Total Trans-p-Hydroxycinnamic Acid Esters

HPLC analysis was performed on a LC-20AT HPLC system (Shimadzu Corporation, Kyoto, Japan) with a SPD-20A UV-VIS detector and a binary pump. The standard solution or test solution (20 μL) was injected onto a Phenomenex Luna C18(2) 100A column (4.6 mm × 250 mm, 5 μm) thermostated at 30 °C. The components were eluted with methanol-0.1% acetic acid aqueous solution (40:60, *v*/*v*) at a flow rate of 1.0 mL·min^−1^ in isocratic mode [21]. The signals at 310 nm were monitored. The results were processed in LabSolutions Analysis Station (Shimadzu Corporation, Kyoto, Japan).

### 3.5. HPLC Validation

The analytical method was validated for specificity, linearity, LOD, LOQ, precision, accuracy, system suitability, and robustness according to the relevant ICH guidelines [24].

#### 3.5.1. Specificity

To assess the specificity of the analytical method and exclude interference from other components in the real samples, the retention times for the real samples and the standard reference were compared. In addition, the resolutions between *trans*-*p*-hydroxycinnamic acid and other compositions in the real samples and the peak purity of *trans*-*p*-hydroxycinnamic acid were determined.

#### 3.5.2. Linearity

The standard solutions of *trans*-*p*-hydroxycinnamic acid at different concentrations (11.0–352.0 μg·mL^−1^) were introduced to the HPLC system in triplicate. The calibration curve of *trans*-*p*-hydroxycinnamic acid was drawn by plotting the peak areas against the corresponding concentrations. The determination coefficient (*r*^2^) of the regression equation was obtained to validate the linearity.

#### 3.5.3. LOD and LOQ

The LOD value was calculated as 3.3 *σ*/*S*, while the LOQ value was calculated as 10 *σ*/*S*, in which *σ* was the residual standard deviation of the regression equation, and *S* was the calibration curve slope.

#### 3.5.4. Precision

In order to validate the precision of the analytical method, the hydrolyzed extract of *L. robustum* at three different concentrations (66.0, 132.0, 264.0 μg·mL^−1^) was used to evaluate the intra-day and inter-day variabilities. The standard solutions were analyzed in triplicate in the same day to obtain the intra-day variability and measured three times in three different days to give the inter-day variability.

#### 3.5.5. Accuracy

In order to validate the accuracy of the analytical method, the recovery of *trans*-*p*-hydroxycinnamic acid was determined by the standard addition method. *Trans*-*p*-hydroxycinnamic acid standard (1.3, 2.6 or 3.9 mg) was added into 1 mL the extracting solution of *L. robustum* which contained 2.64 mg potential *trans*-*p*-hydroxycinnamic acid and then hydrolyzed with sodium hydroxide. The potential *trans*-*p*-hydroxycinnamic acid in the hydrolyzed solution was analyzed by the HPLC method. And the percentage recovery of *trans*-*p*-hydroxycinnamic acid was obtained from the results.

#### 3.5.6. System Suitability

System suitability test was carried out to verify the system, method and column performance. The original extract of *L. robustum* and the hydrolyzed extract of *L. robustum* were analyzed in triplicate. The retention time, resolution, tailing factor, theoretical plates, and injection precision for *trans*-*p*-hydroxycinnamic acid were calculated by LabSolutions Analysis Station.

#### 3.5.7. Robustness

The robustness of the HPLC-UV method was verified by applying minor and deliberate changes in the experimental conditions, including column temperature (30 ± 5 °C), flow rate (1.0 ± 0.2 mL·min^−1^), mobile phase composition (40% ± 2% methanol), and wavelength (310 ± 2 nm) [27]. One parameter was changed at a time by keeping all other parameters at the optimized level. The RSD of the peak area of *trans*-*p*-hydroxycinnamic acid was calculated.

### 3.6. Similarity of HPLC Chromatograms

A newly improved extent similarity method was used to calculate the similarity index of HPLC chromatograms [28], and the similarity of HPLC chromatogram A with HPLC chromatogram B was calculated by following equation:

(2) $$Similarity=1-\sqrt{\frac{\sum_{i=1}^{n}{\left(1-\frac{a_{i}}{b_{i}}\right)}^{2}}{n}}$$

*a_i_*: The peak area of the *i* common peak in HPLC chromatogram A.

*b_i_*: The peak area of the *i* common peak in HPLC chromatogram B.

*n*: The amount of the common peaks in HPLC chromatograms A and B.

### 3.7. Statistical Analyses

Statistical analyses were carried out on GraphPad Prism 5.01. Every sample was determined in triplicate. The results are recorded as mean ± standard deviation (SD). Difference of means between groups was analyzed by one-way analysis of variance (ANOVA) on statistical package SPSS 25.0. The difference between groups was believed to be significant when *p* < 0.05.

## 4. Conclusions

In the present study, a new optimized HPLC-UV method for the quantification of *trans*-*p*-hydroxycinnamic acid and total *trans*-*p*-hydroxycinnamic acid esters in the leaves of *L. robustum* was developed and validated in accordance with ICH guidelines. Because it was difficult and troublesome to analyze no less than 34 *trans*-*p*-hydroxycinnamic acid esters by usual HPLC, these esters were hydrolyzed with sodium hydroxide, and then, the potential *trans*-*p*-hydroxycinnamic acid was determined by HPLC-UV. The above analytical method was simple and rapid, which simplified greatly the analytical process. Additionally, the methodology validation, including specificity, linearity, LOD, LOQ, precision, accuracy, system suitability, and robustness, showed that the new HPLC-UV method was acceptable. To the best of our knowledge, it is the first HPLC method using hydrolyzation for quantification of many carboxylic esters. Finally, the novel method was used successfully to measure the contents of *trans*-*p*-hydroxycinnamic acid and total *trans*-*p*-hydroxycinnamic acid esters in various extracts of the leaves of *L. robustum*. The similarity of the HPLC chromatogram of the original extract of *L. robustum* with the HPLC chromatogram of the hydrolyzed extract of *L. robustum* was 0.103. The method can also be applied to the quality control of the products of *L. robustum*.

## Figures and Tables

**Table 1:** The results of hydrolyzation of *trans*-*p*-hydroxycinnamic acid esters in the extract of *L. robustum*.

Parameter	Condition	Content of Potential *Trans*-*p*-hydroxycinnamic Acid (mg·g^−1^) *^a^*
Catalyst *^b^*	hydrochloride	62.0 ± 0.5 a
	sodium hydroxide	66.0 ± 0.5 b
Incubation temperature (°C) *^c^*	30	46.5 ± 0.4 a
	40	50.1 ± 0.5 b
	50	56.1 ± 0.4 c
	60	59.0 ± 0.5 d
	70	62.5 ± 0.4 e
	80	66.2 ± 0.5 f
	90	66.1 ± 0.5 f
Period of incubation (h) *^d^*	1	62.8 ± 0.5 a
	2	66.0 ± 0.5 c
	4	65.4 ± 0.4 c
	6	64.5 ± 0.5 b

*^a^* Content of potential *trans*-*p*-hydroxycinnamic acid = potential *trans*-*p*-hydroxycinnamic acid weight (mg)/*L. robustum* weight (g). Data are expressed as mean ± standard deviation (*n* = 3). Means with the same letter are not significantly different (one-way analysis of variance, α = 0.05). *^b^* The extracting solution (1 mL) was hydrolyzed with different catalysts (1 mL, 1 M) at 80 °C for 2 h and then neutralized. *^c^* The extracting solution (1 mL) was hydrolyzed with sodium hydroxide (1 mL, 1 M) at different temperatures for 2 h, and then, 1 mL hydrochloride (1 M) solution was added. *^d^* The extracting solution (1 mL) was hydrolyzed with sodium hydroxide (1 mL, 1 M) at 80 °C for different time, and then, 1 mL hydrochloride (1 M) solution was added.

**Table 2:** Results of regression equation, determination coefficient, linear range, LOD, and LOQ for *trans*-*p*-hydroxycinnamic acid by HPLC.

No.	Regression Equation	*r* ^2^	Linear Range (μg·mL^−1^)	Residual STD (*σ*)	Calibration Curve Slope (*S*)	LOD (μg·mL^−1^)	LOQ (μg·mL^−1^)
1	Y = 69.34X + 349.8	1.000	11.0–352.0	51.25	69.34	2.44	7.39
2	Y = 69.58X + 327.1	1.000	11.0–352.0	53.44	69.58	2.53	7.68
3	Y = 69.46X + 347.1	1.000	11.0–352.0	21.83	69.46	1.04	3.14
Integration (*n* = 3)	Y = 69.46X + 341.3	1.000	11.0–352.0	42.17	69.42	2.00	6.07

Limit of detection (LOD) = 3.3 × *σ*/*S*; Limit of quantification (LOQ) = 10 × *σ*/*S*; *σ*, residual standard deviation; *S*, calibration curve slope.

**Table 3:** Intra-day and inter-day variabilities for *trans*-*p*-hydroxycinnamic acid.

Concentration (μg·mL^−1^)	Intra-Day Variability (*n* = 3)		Inter-Day Variability (*n* = 3)	
	Mean ± SD	RSD (%)	Mean ± SD	RSD (%)
66.0	65.4 ± 1.0	1.6	65.2 ± 1.1	1.7
132.0	130.9 ± 1.2	1.0	130.4 ± 1.3	1.0
264.0	262.7 ± 1.3	0.5	261.8 ± 1.3	0.5

RSD, relative standard deviation.

**Table 4:** Results of accuracy validation for *trans*-*p*-hydroxycinnamic acid.

Level	Amount Spiked (mg)	Amount Recovered (mg) *^a^*	Recovery (%) *^a^*
50%	1.3	1.36 ± 0.02	104.6 ± 1.6
100%	2.6	2.68 ± 0.02	103.1 ± 0.8
150%	3.9	3.98 ± 0.03	102.1 ± 0.8
Average (*n* = 9)			103.3 ± 1.1

*^a^* Data are expressed as mean ± standard deviation (*n* = 3).

**Table 5:** System suitability parameters for *trans*-*p*-hydroxycinnamic acid in Figure 2B,C.

Parameter	Figure 2B	Figure 2C	Reference Value
Retention time (min) *^a^*	10.86 ± 0.03	10.88 ± 0.02	-
Resolution *^a^*	2.45 ± 0.10	1.58 ± 0.09	R > 1.5
Tailing factor *^a^*	1.08 ± 0.02	1.29 ± 0.02	≈1
Theoretical plates *^a^*	27,534 ± 439	14,640 ± 257	N ≥ 2000
Injection precision (Area)-%	0.9	0.3	%RSD ≤ 2

*^a^* Data are expressed as mean ± standard deviation (*n* = 3).

**Table 6:** Results of robustness validation for *trans*-*p*-hydroxycinnamic acid.

Parameter	Condition	Peak Area
Column temperature (°C)	25	3407.45
	30	3428.19
	35	3457.14
	Mean	3430.93
	%RSD	0.73
Flow rate (mL·min^−1^)	0.8	3445.89
	1.0	3424.12
	1.2	3408.46
	Mean	3426.16
	%RSD	0.55
Mobile phase composition	38% methanol	3414.56
	40% methanol	3418.79
	42% methanol	3467.14
	Mean	3433.50
	%RSD	0.85
Wavelength (nm)	308	3388.45
	310	3435.36
	312	3370.89
	Mean	3398.23
	%RSD	0.98

**Table 7:** Quantification of *trans*-*p*-hydroxycinnamic acid and total *trans*-*p*-hydroxycinnamic acid esters in the extracts of *L. robustum* depending on extraction solvent.

Sample	Free *Trans*-*p*-hydroxycinnamic Acid (mg·g^−1^) *^a^*	Potential *Trans*-*p*-hydroxycinnamic Acid (mg·g^−1^) *^a^*	Total *Trans*-*p*-hydroxycinnamic Acid Esters (mg·g^−1^) *^a^*	Similarity of Figure 2B with Figure 2C *^b^*
30% EtOH	2.26 ± 0.02 a	37.1 ± 0.7 a	125.8 ± 2.6 a	0.119
40% EtOH	3.34 ± 0.03 b	54.8 ± 0.9 b	185.8 ± 3.3 b	0.101
50% EtOH	3.77 ± 0.02 c	59.9 ± 1.0 c	202.6 ± 3.6 c	0.100
60% EtOH	3.99 ± 0.03 e	62.2 ± 0.8 d	210.1 ± 2.9 c	0.100
70% EtOH	3.96 ± 0.03 e	62.3 ± 1.0 d	210.6 ± 3.6 c	0.099
80% EtOH	3.91 ± 0.02 d	61.5 ± 0.8 d	207.9 ± 2.9 c	0.100
Average				0.103

*^a^* Data are expressed as mean ± standard deviation (*n* = 3). Means with the same letter are not significantly different (one-way analysis of variance, *a* = 0.05). Content of *trans*-*p*-hydroxycinnamic acid = *trans*-*p*-hydroxycinnamic acid weight (mg)/*L. robustum* weight (g). Content of total *trans*-*p*-hydroxycinnamic acid esters = total *trans*-*p*-hydroxycinnamic acid esters weight (mg)/*L. robustum* weight (g). *^b^* $Similarity=1-\sqrt{\frac{\sum_{i=1}^{n}{\left(1-\frac{a_{i}}{b_{i}}\right)}^{2}}{n}}$; *a_i_*, the peak area of the *i* common peak in Figure 2B; *b_i_*, the peak area of the *i* common peak in Figure 2C; *n* = 1, the amount of the common peaks in Figure 2B,C.

## Data Availability

The data presented in this study are available in Supplementary Materials.

## Supplementary Materials

The following supporting information can be downloaded at: https://www.mdpi.com/article/10.3390/molecules28145309/s1, Table S1: The concentrations and peak areas of *trans*-*p*-hydroxycinnamic acid standard; Figure S1: UV spectrum of *trans*-*p*-hydroxycinnamic acid; Figure S2: Calibration curve for *trans*-*p*-hydroxycinnamic acid.

## Sample Availability

Not available.
