# Supplementary material for: A New HPLC-UV Method Using Hydrolyzation with Sodium Hydroxide for Quantitation of Trans-p-Hydroxycinnamic Acid and Total Trans-p-Hydroxycinnamic Acid Esters in the Leaves of Ligustrum robustum
Source: Molecules. 2023 Jul 10;28(14):5309. doi: 10.3390/molecules28145309 (PMC10383156; doi:10.3390/molecules28145309)
Supplement: Supplementary file 1 [file molecules-28-05309-s001.zip › molecules-2479285-supplementary.pdf]

Supplementary information

Table S1. The concentrations and peak areas of *trans*-*p*-hydroxycinnamic acid standard

concentration of *trans*-*p*-hydroxycinnamic acid (μg·mL^{-1})	peak area		
	1	2	3
11.0	1083.37	1012.14	1098.56
22.0	1938.63	1887.41	1901.02
44.0	3428.19	3452.12	3404.62
88.0	6410.12	6446.58	6431.68
176.0	12504.12	12567.25	12589.44
352.0	24784.15	24815.12	24795.74

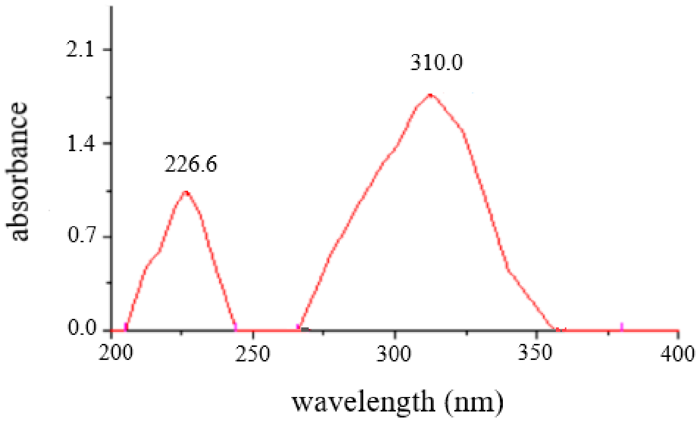

Figure S1. UV spectrum of *trans*-*p*-hydroxycinnamic acid in 40% methanol at 30 °C (12.7 μg·mL^{-1})

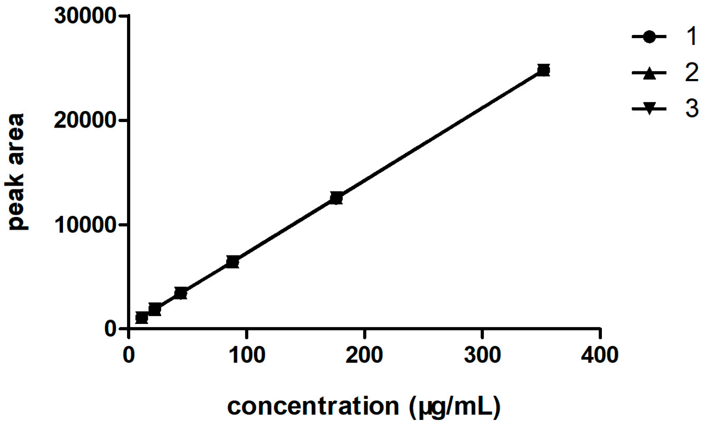

Figure S2. Calibration curve for *trans*-*p*-hydroxycinnamic acid
